# Identification of candidate regulators of the response to early heat stress in climate-adapted wheat landraces *via* transcriptomic and co-expression network analyses

**DOI:** 10.3389/fpls.2023.1252885

**Published:** 2024-01-03

**Authors:** Liam J. Barratt, Sara Franco Ortega, Andrea L. Harper

**Affiliations:** Centre for Novel Agricultural Products (CNAP), Department of Biology, University of York, York, United Kingdom

**Keywords:** heat, transcriptomics, network analysis, *Triticum aestivum*, landrace, hub gene

## Abstract

**Introduction:**

Climate change is likely to lead to not only increased global temperatures but also a more variable climate where unseasonal periods of heat stress are more prevalent. This has been evidenced by the observation of spring-time temperatures approaching 40°C in some of the main spring-wheat producing countries, such as the USA, in recent years. With an optimum growth temperature of around 20°C, wheat is particularly prone to damage by heat stress. A warming climate with increasingly common fluctuations in temperature therefore threatens wheat crops and subsequently the lives and livelihoods of billions of people who depend on the crop for food. To futureproof wheat against a variable climate, a better understanding of the response to early heat stress is required.

**Methods:**

Here, we utilised DESeq2 to identify 7,827 genes which were differentially expressed in wheat landraces after early heat stress exposure. Candidate hub genes, which may regulate the transcriptional response to early heat stress, were identified via weighted gene co-expression network analysis (WGCNA), and validated by qRT-PCR.

**Results:**

Two of the most promising candidate hub genes (TraesCS3B02G409300 and TraesCS1B02G384900) may downregulate the expression of genes involved in the drought, salinity, and cold responses—genes which are unlikely to be required under heat stress—as well as photosynthesis genes and stress hormone signalling repressors, respectively. We also suggest a role for a poorly characterised sHSP hub gene (TraesCS4D02G212300), as an activator of the heat stress response, potentially inducing the expression of a vast suite of heat shock proteins and transcription factors known to play key roles in the heat stress response.

**Discussion:**

The present work represents an exploratory examination of the heat-induced transcriptional change in wheat landrace seedlings and identifies several candidate hub genes which may act as regulators of this response and, thus, may be targets for breeders in the production of thermotolerant wheat varieties.

## Introduction

The damaging effect of heat stress exposure on *Triticum aestivum* L. (bread wheat) yields is well known, with reductions between 3% and 6% being observed for every degree increase above the crop’s optimal growth temperature of 20°C (Chowdhury and Wardlaw, 1978; Kobza and Edwards, 1987; Wardlaw et al., 1989; Nagai and Makino, 2009; Ray et al., 2013; Zhao et al., 2017; Tian et al., 2018), with such heat-induced yield losses being evidenced in recent field trials (Riaz et al., 2021; Roychowdhury et al., 2023; Wang et al., 2023). These kinds of yield losses are likely to become more common in the coming years as a result of climate change and global warming, as, according to Intergovernmental Panel on Climate Change (IPCC) predictions, an increase in global mean surface temperatures of between 0.3°C and 4.8°C, compared with the prior century, are expected by 2100 (Collins et al., 2013), whereas other models predict more rapid global temperature increases, with such levels being reached by the year 2060 (Wigley and Raper, 2001; Murphy et al., 2004; De Costa, 2011). This is particularly worrying considering that global wheat consumption in 2021/2022 reached almost 800 million metric tonnes and currently accounts for 20% of the globe’s annual calorie consumption (Pfeifer et al., 2014; Food and Agriculture Organization of the United Nations et al., 2018; United States Department of Agriculture - Foreign Agricultural Service, 2023), meaning the lives, and livelihoods, of billions around the world depend on the success of the yields of this single crop.

Not only are yearly average global temperatures rising, but seasonal temperature patterns are likely to shift over the coming years, with warmer springs already being increasingly reported; for example, 10 of the 13 springs to have occurred since 2010 make up the warmest springs ever recorded, with spring 2022 ranking sixth on this list (NOAA National Centers for Environmental Information, 2022a). This trend toward increasingly warm spring months saw much of Western, Southern, and Central Europe experience record temperatures in May 2022, whereas Southern states of the USA experienced their fourth warmest May since records began in 1895 (NOAA National Centers for Environmental Information, 2022a; NOAA National Centers for Environmental Information, 2022b). Not only are average temperatures during the spring months rising globally, but unseasonal periods of extreme temperatures are expected to occur more frequently as the climate continues to change (Easterling et al., 2000; Thornton et al., 2014; Haokip et al., 2020). Evidence of such events have been observed recently, with the USA, Italy, and Turkey all experiencing periods of elevated temperature, above 30°C, in May 2020 (NOAA National Centers for Environmental Information, 2020), whereas parts of the American Midwest, such as major spring-wheat-producing state Minnesota, saw temperatures reach 38°C (NOAA National Centers for Environmental Information, 2018).

This is a pressing issue for much of the Northern Hemisphere, in addition to South Asia and the Middle East, as, in many western countries, spring wheat is often sown during March and April. The springtime sowing of seeds in these regions means warmer spring months, and increasingly prevalent periods of extreme temperatures coincide with the early vegetative development of spring wheat crops in regions of high production, such as the USA, Canada, and the United Kingdom. These countries produced over 80 million tonnes of wheat combined in 2021 (Food and Agriculture Organization of the United Nations, 2023) and so play crucial roles in global food security. Therefore, it is essential that spring wheat crops in such countries are protected against the increasingly likely threat of heat stress during early development.

The first step toward achieving this is to gain a better understanding of both thermotolerance and the heat stress response during early vegetative development. Having previously identified a candidate master hub, and three validated genetic markers, for early basal thermotolerance (Barratt et al., 2023a), the present work builds on this previous experiment, this time aiming to understand the transcriptional response to early heat stress in spring habit wheat landraces and identify candidate hub genes, which may regulate this response using weighted gene co-expression network analysis (WGCNA). Together, these works provide a comprehensive examination of early heat stress exposure in bread wheat, generating insights into how these processes may be regulated transcriptionally, and identifying genes which may be responsible for this regulation.

A handful of studies have examined the effect of heat stress on the transcriptome of wheat during vegetative development (Qin et al., 2008; Liu et al., 2015; Jin et al., 2020); however, this type of analysis paired with subsequent network analysis is less common, despite this approach enabling the identification of a small number of promising candidate genes potentially playing large regulatory roles in the stress response, reducing the time spent laboriously screening all of the identified stress-responsive genes. Similar combined approaches have been used in other contexts, however, such as to identify regulators of thermotolerance during vegetative development in wheat (Girousse et al., 2018; Mishra et al., 2021); response to heat and cold stresses, and basal thermotolerance in rice (Wang et al., 2022; Zeng et al., 2022; Boulanger et al., 2023); response to combined heat, drought, and salinity stresses in Brachypodium (Shaar-Moshe et al., 2017); and drought stress response in sugarcane (Tang et al., 2023), whilst we have previously used this combined approach to study the drought stress response in wheat (Barratt et al., 2023b). However, there are no similarly exploratory examples of this approach being used to study the heat stress response in wheat, as yet. Although one study has utilised similar approaches to identify genes that may be regulated by preexisting candidate genes under heat stress, by examining the effect of heat stress exposure on knockout mutants and wild-type plants (Tian et al., 2022), our study represents the first exploratory example of WGCNA utilisation to identify candidate hub genes which may regulate the heat stress response during vegetative development in climate-adapted bread wheat landraces.

## Materials and methods

### Plant growth conditions

The seeds used in the present work were from plants which derived from at least three generations of selfing. There were 13 accessions previously shown to be distinctively tolerant or susceptible under heat stress (Barratt et al., 2023a; **Supplementary Data Sheet 1**) sown in Levington Advance Seed & Modular F2S compost mixed with Aggregate Industries Garside Sands 16/30 sand in an 80:20 ratio, which was treated with Calypso insecticide (Bayer Crop Science Ltd., 0.083 ml mixed with 100 ml water, applied to each litre of compost). The heat stress treatment used in the present work was identical to that used previously (Barratt et al., 2023a), as it was found to significantly disrupt plant growth, without being lethal. Plants were placed into a Percival AR-75L growth cabinet with 18-h day length and respective day/night temperatures of 22°C and 16°C until the three-leaf stage. At this point, four replicates of each accession were transferred to a separate Percival AR-75L growth cabinet and exposed to 35°C/30°C (day/night) for 14 days, with all other conditions being the same as in the control cabinet. Both control and heat-stressed plants were well-watered, ensuring that the compost was kept moist with daily watering. 2-cm leaf tissue samples taken at the three-leaf stage and after 14 days of heat stress exposure were frozen in liquid nitrogen and stored at −80°C prior to RNA extraction.

### RNA extraction, sequencing, and mapping

Leaf tissue samples weighing less than 100 mg were used for total RNA extraction *via* the E.Z.N.A Plant RNA Kit (Omega Bio-Tek, GA, USA) including a DNase treatment, according to the manufacturer’s protocol. Both NanoDrop ND-1000 Spectrophotometer (Thermo Fisher Scientific, MA, USA) and Qubit 4 Fluorometer (Life Technologies, CA, USA) were used for quantification of RNA concentration, whereas Agilent Technology 2100 Bioanalyzer (Agilent Technologies, CA, USA) was used to assess RNA quality. Samples were deemed to be acceptable for use in subsequent analysis if their RNA Integrity Number (RIN) value was >7. To help control the effect of the environment on the transcriptome, prior to sequencing, we pooled acceptable RNA from at least three replicate plants per accession, per condition (pre- or post-heat stress), whereas biological replication for each treatment was provided by the different accessions. Samples were stored at −80°C and shipped on dry ice to Novogene (Cambridge, United Kingdom) for sequencing using the Illumina NovaSeq 6000 platform (Illumina, CA, USA) with a 150-bp paired-end sequencing strategy. Raw reads were trimmed using Trimmomatic v0.39 (Bolger et al., 2014) by removing leading and trailing low-quality or N bases (below quality 3), minimum length 36 bp and sliding window 4:15. FastQC (www.bioinformatics.babraham.ac.uk/projects/fastqc/) was used to assess the quality of the data, then Salmon v0.8.1 (Patro et al., 2017) was used to map trimmed reads to the *Triticum aestivum* reference genome v1.1 (IWGSC RefSeq v1.1, http://ftp.ensemblgenomes.org/pub/plants/release-46/fasta/triticum_aestivum/). Salmon transcripts per million (TPM), counts, and lengths were inputted into R (version 4.1.2.; R Core Team, 2021) using TxImport (Soneson et al., 2015) for further analysis.

### Transcriptomic and differential expression analyses

There were 26 pooled RNA samples from 13 accessions (before and after heat stress, for 13 accessions) used for transcriptomic analysis. After importing transcriptome data into R using TxImport, the principal component analysis (PCA) function of DESeq2 (version; 1.36.0; Love et al., 2014) was first used to explore count data from RNA-Seq. Genes with fewer than 10 non-zero entries were then removed, leaving 75,732 genes for differential expression analysis (DEA; **Supplementary Data Sheet 2**). DEA was carried out using the DESeq2 package (version 1.36.0; Love et al., 2014) in R, whereby an additive model was used to identify genes differentially expressed between tolerant and susceptible accessions, as well as between pre- and post-stress samples. To make our selection of DEGs robust to replicate variability, we used the adaptive shrinkage function (ashr) to shrink effect sizes of genes with high dispersion values. Genes which showed a log2FC above/below 1.5/−1.5 and an FDR-adjusted *p*-value (Benjamini and Hochberg, 1995) below, or equal to, 0.05 for either of these comparisons were deemed to be tolerance or response differentially expressed genes (DEGs), respectively. The adaptive shrinkage function was employed in the ranking of genes to shrink the log fold-change estimates of genes with low counts or high dispersion (Stephens, 2017). Due to extremely low numbers of tolerance DEGs identified in the DEA, only response DEGs were studied further.

### GO term enrichment analysis

To identify gene ontology (GO) terms significantly enriched amongst upregulated and downregulated response DEGs identified *via* DEA, GO enrichment analysis was conducted. An approach, used previously (Borrill et al., 2019; Andleeb et al., 2023), was adopted to transfer GO terms to the v1.1 annotation, from the IWGSC RefSeqv1.0 genome annotation, as GO terms are only available for the v1.0 annotation. The list of genes for which GO terms can be transferred can be found in Andleeb et al. (2023). IWGSC v1.0 GO terms were read into R using the base R function readRDS() for analysis, after being retrieved from https://opendata.earlham.ac.uk/wheat/under_license/toronto/Ramirez-Gonzalez_etal_2018-06025-Transcriptome-Landscape/data/TablesForExploration/FunctionalAnnotation.rds.

GO terms that upregulated and downregulated response DEGs are annotated with were then collated into two groups, before the agriGO Singular Enrichment Analysis tool (Du et al., 2010; Tian et al., 2017) was used to conduct a Fisher’s exact test on both groups of GO terms, with the GO terms of all genes included in DEA serving as background. 0.05 was the *p*-value threshold; Hochberg (FDR) was the multi-test adjustment method (Benjamini and Hochberg, 1995), and 5 was the minimum number of mapping entries threshold. Significantly enriched GO terms had an FDR-adjusted *p*-value < 0.05. AgriGO’s DAG Drawer tool was also used to generate DAG trees for significantly enriched GO terms.

### Network construction and module detection

**Figure 1:**
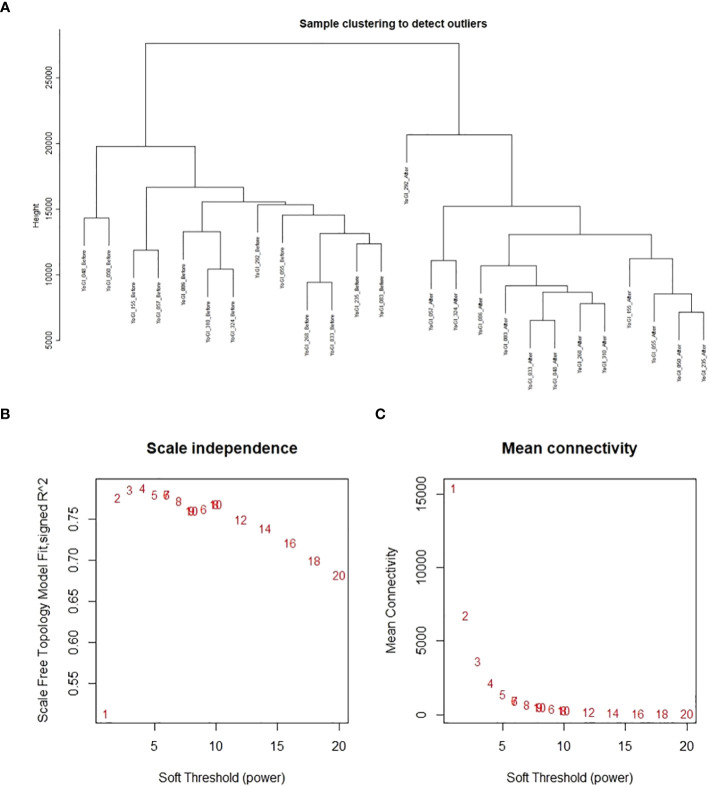
Analyses performed by WGCNA prior to co-expression network construction. Results of sample clustering **(A)**, scale-free topology fit index as a function of the soft-thresholding power **(B)**, and mean connectivity as a function of the soft-thresholding power **(C)**.

A single co-expression network was constructed *via* the WGCNA R package (Langfelder and Horvath, 2008; Langfelder and Horvath, 2012), using TPM data provided by Salmon. No samples were removed after clustering, but 19,965 genes were removed due to too many zero values: leaving 87,580 genes from 26 samples (13 accessions, before and after heat stress exposure) for network construction. The blockwiseModules() function conducted blockwise network construction according to the function’s default parameters, except the following: network type = signed hybrid, maximum block size = 5000, soft threshold power = 8 (advised by the package’s authors for this number of samples, as no soft threshold power exceeded a reasonable scale-free topology fix index of 0.8), minimum module size = 30, merge cut height = 0.25. After module detection, edge and node files were created using the “exportNetworkToCytoscape()” function with a threshold of 0.1; filtering out weak connections between genes (nodes). Results of sample clustering, scale-free topology fit index as a function of the soft-thresholding power, and mean connectivity as a function of the soft-thresholding power can be found in **Figure 1**. Gene expression data after sample clustering and processing *via* WGCNA, and network construction data are available on GitHub: https://github.com/andreaharper/HarperLabScripts/.

### Identifying stress-associated modules

To understand the likely functions of genes within each module, GO enrichment analysis was conducted using the same approach as outlined above. Here, however, GO terms associated with genes within a module were collated and submitted to the agriGO Singular Enrichment Analysis tool, with the GO terms of all genes included in the network serving as background. All other parameters were the same as described above.

As well as this, we also conducted DEG enrichment analysis to identify which modules in the co-expression network contained a significantly larger proportion of response DEGs than expected and thus may be particularly associated with the heat stress response. To test whether a module was significantly enriched in response DEGs (observed proportion of DEGs above 8.94%), a one-proportion Z-test was used. Modules were deemed to be significantly enriched in DEGs if *p* was < 0.05.

### Network visualisation and hub identification

Degree (connection) scores were calculated for each gene, *via* either the Cytoscape network analyser tool (Assenov et al., 2008), after first visualising network modules in Cytoscape (version 3.9.1.; Shannon et al., 2003), or *via* counting the number of connections to and from each gene in the module’s WGCNA edge file using the table() function in R. The script used to calculate degree scores in R is available on GitHub (https://github.com/andreaharper/HarperLabScripts/). Visualisation and analysis in Cytoscape were used to identify hub genes in the majority of the modules; however, the edge counting method in R was used to calculate degree scores for genes in particularly large modules (containing more than ~2,000 genes), which often cannot easily be loaded, viewed, and analysed in Cytoscape. For the largest modules, the R package vroom (version 1.6.3.; https://vroom.r-lib.org) was used to read Cytoscape edge files into R for analysis.

We selected hub genes for further analysis based on their high degree scores, significant levels of differential expression, and annotated functions with potential regulatory roles. In cases where multiple genes within a module shared the highest degree score, or the highest degree-scoring genes were found not to be differentially expressed under heat stress, the highest-scoring DEG was deemed to be the hub gene. These well-connected DEGs were selected for further enquiry as they were deemed to be more likely to act as coordinators of the transcriptional response to heat stress than well-connected genes that were not differentially expressed. Where the putative function of the most well-connected DEG suggested no involvement in either the control of gene expression (be that directly as a transcription factor, or more indirectly *via* involvement in signalling pathways), or in the heat stress response/thermotolerance (for example, as a heat shock protein; HSP), other DEGs with similar degree scores, which were predicted to play such roles based on their annotation, were favoured as the candidate hub gene. If no such well-connected DEGs within a module were likely involved in such processes, the most well-connected DEG was deemed to be the module’s hub gene. Uncharacterised hub genes were studied further, as they represented novel candidates for potential regulators of the heat stress response. Orthologues of hub genes, and genes they were connected to, were identified *via* Ensembl Plants (Yates et al., 2022).

### qRT-PCR

cDNA was obtained from the RNA extracted for each one of the biological replicates of the 13 landraces (4 biological replicates, 2 treatments) of the mRNA-Seq experiment. The reaction was carried out using the ImProm-II™ Reverse Transcription System (Promega) using the manufacturer’s instruction, 1 µl of Oligo(dT)16 (5 µM) (Eurogentec Ltd, Camberley, UK) and 1µl of each RNA sample. Quantification was performed with a Nanodrop 2000 and after that, the cDNA of the 4 biological replicates were pooled in equimolar concentrations. qRT-PCR was performed for the genes TraesCS1B02G384900, TraesCS3B02G409300 and TraesCS4D02G212300 and tubulin using the primers described in Supplementary Data Sheet 7 and the iTaq Universal SYBR Green Supermix (Bio-Rad), adding 200ng of cDNA and 0.1 µM of each primes. The qRT-PCR protocol was set on QuantStudio™ 7 Pro Real-Time PCR System (ThermoFisher) as follows: 95 °C for 4.5 min, 40 cycles of 95°C for 15s and 60°C for 15s. The melting curve was performed by initially heating in a 4.5°C/s ratio up to 95°C and maintaining for 10s reducing the temperature to 3.44°C/s up to 60°C and heating in a 0.15°C/s to 95°C kept for 10s with fluorescence measurement in the last step of the PCR and melting curve. The relative expression between after- and before-heat samples was calculated using the delta-delta Ct method using tubulin as the reference.

## Results

### Transcriptome sequencing and quantification

**Figure 2:**
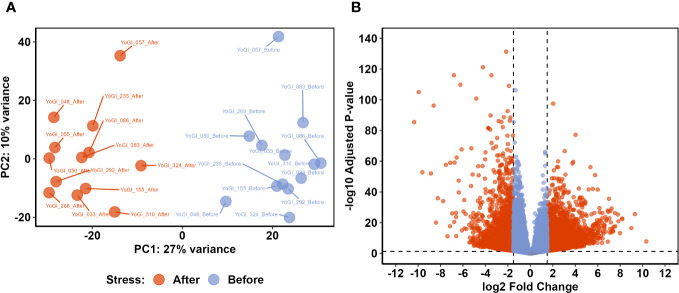
Comparative transcriptomic analysis identified a shift in the wheat transcriptome after exposure to early heat stress. Principal component analysis (PCA) of variance-stabilised counts from all 26 samples **(A)** showed clear separation between the two groups on PC1. Differential expression analysis identified 7,827 DEGs with significantly different expression before and after exposure to early heat stress **(B)**. Dashed lines indicate DEG thresholds: vertical lines represent the log2FC thresholds of ±1.5, whereas horizontal lines represent the *p*-value threshold of 0.05. DEGs which met these criteria are beyond these threshold lines, coloured red.

An average of 49,798,391 reads were obtained from each sample (minimum of 39,938,210 and maximum of 60,591,112) with an average of 92.5% of reads with Q30 and a GC content of 54.9. After trimming, an average of 2,888,493 reads were kept for each sample. An average of 71.5% of the trimmed reads mapped against the wheat genome. Raw data and Salmon outputs are publicly available in the Gene Ontology Repository (accession number: GSE23236). DESeq2 was used to variance-stabilize counts from all 26 samples, before the 500 most variable genes were assessed *via* principal component analysis (PCA; **Figure 2A**). PC1 and PC2, combined, explained 37% of the total variance, with clear distinction between samples taken before and after heat stress exposure being apparent on PC1 (which explained 27% of the observed variation). PC2 explained a smaller proportion of the total variation (10%) and provided some separation between samples, likely relating to variation in each accession’s geographical point of origin.

### Identification of DEGs and comparative transcriptomic analysis

To identify genes which may be involved in the heat stress response and basal thermotolerance, we employed DEA *via* DESeq2 (Love et al., 2014). The analysis identified 7,827 genes which were significantly differentially expressed before and after heat stress exposure (response DEGs; **Supplementary Data Sheet 3**), as well as 93 genes which were differentially expressed between tolerant and susceptible accessions (tolerance DEGs; **Supplementary Data Sheet 3**). Of the response DEGs, 5,384 were significantly upregulated after heat stress exposure, whereas 2,443 were significantly downregulated (**Figure 2B**). There were 41 tolerance DEGs expressed at significantly higher levels in tolerant accessions, whereas 52 tolerance DEGs were expressed more in susceptible accessions. The total number of tolerance DEGs was almost 100-fold less than the total number of response DEGs, and so response DEGs became the main point of inquiry in the subsequent analyses.

To understand the likely functionalities of the genes differentially expressed under heat stress, and to examine the differences in gene functionalities between these groups, we conducted GO enrichment analysis on the two DEG groups (**Supplementary Figure 1**; **Supplementary Data Sheet 4**). We found that GO terms related to DNA damage and replication [for example; “DNA integrity checkpoint” (GO:0031570), “DNA damage checkpoint” (GO:0000077), and “DNA replication” (GO:0006260)] were significantly enriched amongst upregulated DEGs, as was the term “protein refolding” (GO:0042026). We also found an abundance of terms related to cell wall processes [for example; “Cell wall assembly” (GO:0070726) and “Plant-type cell wall organization or biogenesis” (GO:0071669)], as well terms related to both cellulose [for example; “Cellulose microfibril organization” (GO:0010215) and “Cellulose biosynthetic process” (GO:0030244)] and lignin [for example; “Lignin metabolic process” (GO:0009808) and “Phenylpropanoid metabolic process” (9.7e-05)] synthesis and organization.

However, amongst downregulated DEGs, terms related to photosynthesis were significantly enriched, for example: “Photosynthesis” (GO:0015979), “Photosynthesis, light reaction” (GO:0019684) and “Photosynthetic electron transport in photosystem II” (GO:0009772). Terms related to the drought response were also significantly enriched, for example: “Response to water” (GO:0009415) and “Trehalose biosynthetic process” (GO:0005992), as were terms related to the general stress response, for example: “Response to stress” (GO:0006950), “Response to oxidative stress” (GO:0006979) and “Response to stimulus” (GO:0050896). Similarly, terms potentially related to the salinity response, for example: “Ion transport” (GO:0006811), “Cation transport” (GO:0006812), “Ion homeostasis” (GO:0050801) and “Sodium ion transport” (GO:0006814) were also significantly enriched amongst downregulated DEGs.

### Identifying stress-associated modules

The co-expression network was consisted of 73 modules (**Supplementary Data Sheet 5**), housing 87,580 genes. Modules within the co-expression network ranged in size from 36 to 26,420 genes, whereas mean and median module size were 1,120 and 310 genes, respectively.

Modules significantly enriched in the “response to heat” (GO:0006951), “response to temperature stimulus” (GO:0009266) or “response to stress” (GO:0006950) GO terms likely contain genes involved in the response to heat stress. We found that 11 modules were significantly enriched in these, or other stress-associated, GO terms (**Table 1**), with the turquoise and yellow modules being significantly enriched, specifically, in the “response to heat” GO term. Although it was enriched in the “response to water” (GO:0009415) GO term, the black module may also contain genes involved in responding to elevated temperatures, as drought stress often occurs simultaneously with heat stress. The most significantly enriched GO term, and any significantly enriched stress-associated GO terms, in each module can be seen in **Supplementary Data Sheet 6**.

**Table 1:** A total of 11 modules significantly enriched in GO terms related to the stress response, according to GO enrichment analysis by the AgriGO v2.0 Singular Enrichment Analysis tool (Du et al., 2010; Tian et al., 2017).

Module	Enriched GO term	FDR-adjusted *p*-value
Black	Regulation of RNA biosynthetic process Response to water	6.9E-24 0.02
Brown	Amino sugar catabolic process Response to oxidative stress	9E-15 1.3E-10
Darkgreen	Multi-multicellular organism process Response to stress	9.5E-13 0.001
Darkolivegreen	Sexual reproduction Response to oxidative stress	0.0001 0.008
Green	Response to oxidative stress	1.2E-09
Lightcyan	Nucleosome organisation Response to stress	3.4E-09 0.03
Pink	Photosynthesis Cellular response to stimulus	5.8E-18 0.001
Red	Response to biotic stimulus Response to stress	3.6E-14 1.7e-05
Salmon	Protein phosphorylation Response to stress	5.9E-08 0.002
Turquoise	Translation Response to heat	9E-128 0.02
Yellow	Cellular protein localisation Response to heat	1.3E-29 0.02

The modules enriched in such GO terms are listed, as well as the most significantly enriched GO term, and the stress-associated GO term they were also enriched in, respectively. In the instances where stress-associated GO terms were the most significantly enriched term in a module, only that term is given. The FDR-adjusted Fisher exact test p-values associated with each enriched GO term are given in brackets.

To further explore which modules may be associated with the heat stress response, we aimed to identify modules significantly enriched in response DEGs, *via* DEG enrichment analysis. We found that 11 modules were significantly enriched in response DEGs (*p* < 0.05), with a one-proportion Z-test identifying that the proportion of DEGs in these modules was significantly greater than the expected proportion of 8.94% (**Table 2**). Amongst these modules, the observed proportions of DEGs ranged from 11.4% (turquoise) to 39.9% (pink).

**Table 2:** A total of 11 modules were significantly enriched in DEGs, as they contained a significantly higher proportion of DEGs than expected should the total number have been distributed across modules according to their size (8.94%).

Module	Number of genes	Observed percentage of DEGs	Mean log2FC of DEGs	*p*-value
Black	2,415	17.8	-2.37	1.82E-52
Blue	6,821	14.2	2.54	6.34E-52
Darkgrey	676	29	-2.42	6.61E-75
Darkseagreen4	96	22.9	2	7.95E-07
Darkslateblue	155	25.8	-1.92	9.22E-14
Floralwhite	169	27.2	-2.13	4.10E-17
Grey60	1,360	21	-2.19	1.07E-54
Pink	2,202	39.9	-2.4	0
Plum	58	34.5	-2.94	4.62E-12
Thistle1	128	13.3	-2.53	0.04
Turquoise	26,420	11.4	2.33	6.15E-45

These modules are listed, as well as the number of genes in each module, the percentage of these genes which were observed to be DEGs, the mean log2 fold change of the DEGs within each module, and the p-value result from the one-proportion Z-test.

### Hub gene identification

**Figure 3:**
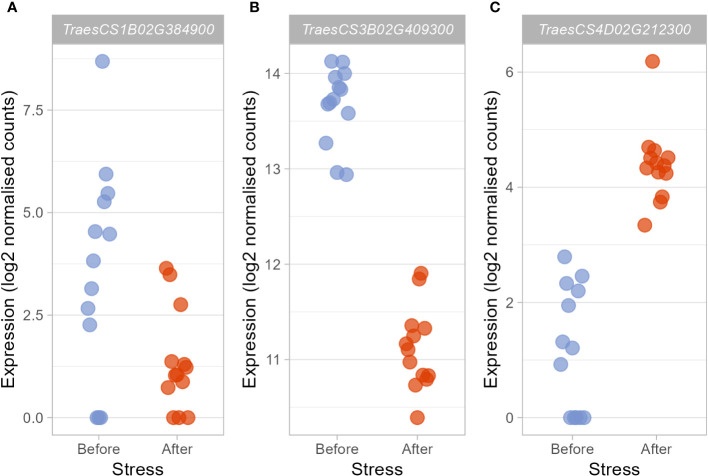
Candidate hub genes which may regulate the heat stress response were differentially expressed after heat stress exposure. Those hub genes deemed to be particularly promising based on their membership within stress-associated modules, their putative function, and the putative functions of the DEGs they were connected to showed varying responses to heat stress. *TraesCS1B02G384900* **(A)** and *TraesCS3B02G409300* **(B)** were significantly downregulated (log2FC = −2.98 and −2.52, respectively), whereas expression of *TraesCS4D02G212300* **(C)** was significantly upregulated (log2FC = 3.29).

Within these stress-associated modules, determined either due to an enrichment of stress-associated GO terms (**Table 1**) or an enrichment of DEGs (**Table 2**), well-connected DEGs were identified as hub genes which may act to coordinate the transcriptional response to early heat stress. These hub genes are seemingly involved in a range of processes, from thermotolerance, to stress hormone signalling and photosynthesis (**Table 3**). However, three of these hub genes, in particular (*TraesCS1B02G384900*, *TraesCS3B02G409300*, and *TraesCS4D02G212300*; **Figure 3**), were deemed to be the most promising candidate hub genes, not only potentially regulating the transcriptional heat stress response (like the other hub genes) but also the physiological heat stress response—thanks to their own likely function, and the likely functions of the genes they are connected to in their respective modules. Both *TraesCS1B02G384900* and *TraesCS3B02G409300* may determine the expression of potentially superfluous genes, as well as the expression of stress hormone signalling repressors and photosynthesis genes, respectively. *TraesCS4D02G212300*, however, may coordinate the expression of a vast suite of heat shock proteins (HSPs), small heat shock proteins (sHSPs), and stress-responsive transcription factors.

**Table 3:** Hub genes identified in stress-associated modules may be strong candidates for regulators of the heat stress response, based on their high number of connections to other genes within stress-associated modules.

Module	Hub Gene	Log2FC	BLAST Hit	Putative function	Reference
Black	*TraesCS1B02G384900*	-2.98	*T. aestivum mitogen-activated protein kinase kinase kinase 18-like*	ABA signal transduction	(Matsuoka et al., 2015)
Blue	*TraesCS4D02G212300*	3.29	*T. aestivum 17.9 kDa class I heat shock protein-like (LOC123097951)*	Thermotolerance	(Chauhan et al., 2012)
Brown	*TraesCS4B02G118900*	2.2	*T. aestivum peroxidase 4-like*	ROS homeostasis, and lignification	(Fernández-Pérez et al., 2015)
Darkgreen	*TraesCS2D02G589600*	1.65	*TaGSTU153*	Uncharacterised	(Wang et al., 2019)
Darkgrey	*TraesCS1D02G205700*	-1.74	*T. aestivum probable protein phosphatase 2C 47/AtPP2CG1*	Abiotic stress response	(Liu et al., 2012)
Darkolivegreen	*TraesCS4D02G364400*	-3.82	*T. aestivum transcription factor GHD7-like*	Nitrogen utilisation, and regulation of flowering time	(Zheng et al., 2019; Wang et al., 2021)
Darkseagreen4	*TraesCS5B02G145800*	2.38	*T. aestivum chalcone isomerase-like protein 2*	Flavonoid synthesis	(Waki et al., 2020)
Darkseagreen4	*TraesCS5D02G145400*	1.62	*T. aestivum chalcone isomerase-like protein 2*	Flavonoid synthesis	(Waki et al., 2020)
Darkslateblue	*TraesCS7D02G333900*	-1.67	*T. aestivum phosphoenolpyruvate carboxylase Tappc1bD*	Photosynthesis and respiration	(Mazelis and Vennesland, 1957)
Floralwhite	*TraesCS1A02G298600*	-2.03	*T. aestivum WRKY transcription factor WRKY24-like*	Stress hormone signalling repression	(Zhang et al., 2015)
Green	*TraesCS5D02G268900*	1.88	*TaGSFT81*	Uncharacterised	(Wang et al., 2019)
Grey60	*TraesCS2A02G357800*	-1.67	*T. aestivum calmodulin-binding receptor-like cytoplasmic kinase 3*	Calcium signalling	(Zeng et al., 2015)
Lightcyan	*TraesCS1B02G221100*	-2.34	*T. aestivum silicon efflux transporter LSI3-like*	Silicon homeostasis	(Yamaji et al., 2015)
Pink	*TraesCS3B02G409300*	-2.52	*T. aestivum protein EARLY RESPONSIVE TO DEHYDRATION 15-like*	ABA signalling repression	(Kariola et al., 2006)
Plum	*TraesCS4D02G298300*	-4.18	*Aegilops tauschii subsp. strangulata Bowman-Birk type trypsin inhibitor*	Protease inhibition	(Gitlin-Domagalska et al., 2020)
Red	*TraesCS7A02G147300*	5.15	*T. aestivum bidirectional sugar transporter SWEET15-like*	Regulation of cell viability under stress	(Seo et al., 2011)
Salmon	*TraesCS1B02G164200*	-1.59	*T. aestivum hypersensitive-induced response protein 1-like*	Hypersensitive response	(Zhou et al., 2010)
Thistle1	*TraesCS5A02G454200*	-1.81	*T. aestivum chlorophyll a-b binding protein of LHCII type 1-like*	Photosynthesis, and ABA signalling	(Liu et al., 2013)
Turquoise	*TraesCS1D02G061400*	1.94	*T. aestivum pentatricopeptide repeat-containing protein At1g09900-like*	Uncharacterised	
Yellow	*TraesCS4B02G339800*	4.2	*T. aestivum sugar transporter ERD6-like 4*	Response to water, and Sugar transport	(Kiyosue et al., 1998)

Each hub gene’s module membership and log2FC are given, as well as their identity and putative function.

### qRT PCR validation

qRT-PCR confirmed the patterns of expression for TraesCS3B02G409300 (t-test, *t*(24)=5.09, *p*<0.0001, *n*=26) and TraesCS4D02G212300 (t-test, *t*(19.83)=-6.56, *p*<0.0001, *n*=26), which were found to be down- and up-regulated respectively after heat stress, supporting the role of these genes in activating the early heat stress response. However we were not able to confirm the down-regulation of TraesCS1B02G384900 by qRT-PCR (t-test, *t*(23.59, *p*=0.98, *n*=26).

## Discussion

### Heat stress causes widespread changes in the wheat transcriptome

In the present work, we demonstrate that the expression profiles of almost 8,000 genes in the spring wheat transcriptome are significantly altered by exposure to early heat stress; 5,384 and 2,443 genes being significantly upregulated and downregulated, respectively. Amongst these groups of DEGs, genes with different functionalities were significantly enriched. Perhaps predictably given their importance as part of the heat stress response, genes involved in protein refolding were enriched amongst upregulated DEGs (Wang et al., 2004; Kotak et al., 2007; Al-Whaibi, 2011; Mogk et al., 2018; Tian et al., 2021). As well as disrupting protein homeostasis, periods of elevated temperature will also cause single- and double-stranded breaks in DNA, whilst also halting the progression of the replication fork (Velichko et al., 2012; Kantidze et al., 2016; Han et al., 2021). The need to protect cells against such heat-induced DNA damage is a key part of the heat stress response, shown previously to increase thermotolerance in Arabidopsis (Han et al., 2020) and evidenced by the enrichment of GO terms related to DNA replication and repair amongst upregulated DEGs. Similarly, we observed the enrichment of GO terms related to cell wall processes and lignin biosynthesis—likely evidence of the cell wall remodelling known to occur in plants during periods of high temperature (Yang et al., 2006; Lima et al., 2013; Le Gall et al., 2015; Wu et al., 2018; Pinski et al., 2021), with lignin synthesis being identified as an important thermotolerance mechanism in rice (Cai et al., 2020).

Amongst downregulated DEGs, however, we saw the significant enrichment of many terms related to photosynthesis, and photosystem II (PSII) in particular. PSII is particularly vulnerable to damage by heat stress (Yamamoto, 2016; Wang et al., 2018; Hu et al., 2020); therefore, the abundance of these genes amongst downregulated DEGs suggests a partial shutdown of PSII and, thus, a reduced photosynthetic rate under heat stress. Interestingly, we saw the enrichment of terms related to the general stress response amongst downregulated DEGs, suggesting these genes play no role in the tailored heat stress response. Perhaps related to this, we also found that terms related to the response to drought and salinity were enriched amongst downregulated DEGs, including the orthologue of AtPP2CG1, which responds to abscisic acid and positively regulates salt stress tolerance in Arabidopsis (Liu et al., 2012). It may be possible, therefore, that these genes are downregulated to increase transcriptional capacity for genes involved directly in the heat stress response. Similar widespread downregulation of drought- and salinity-responsive genes under heat stress has not been extensively described previously in similar works in wheat (Qin et al., 2008; Rangan et al., 2020; Azameti et al., 2022; Lee et al., 2022). Nor was a comparable shift seen when we examined the effect of early drought stress on the wheat transcriptome (Barratt et al., 2023b); for example, only 161 (2.99%) of the 5,384 upregulated heat-responsive DEGs identified in the present work were also downregulated under early drought stress in our previous work, whereas almost double this number of upregulated drought DEGs (321) were downregulated in the present work after heat stress exposure.

This perhaps speaks to the similarity of the different abiotic stresses, as although drought, salinity, freezing, and heat stresses all cause damage to protein structure, functionality, and cell membrane stability, there exist stress-specific cellular environments under drought, salinity, and freezing stresses that are not observed in well-watered plants exposed to high temperatures—such as desiccation, ion imbalance, and ice crystal formation. It is those genes involved in responding to these specialised cellular environments, therefore, that are likely to be superfluous under heat stress and, subsequently, are also likely to be downregulated. The same cannot be said for many of the genes involved in responding to heat stress, however, as these genes are largely involved in mitigating the effects common amongst all the abiotic stresses, particularly damage to proteins and membranes. The relatively large crossover potential of such genes in responding to different abiotic stresses, as a result of the similarities in cellular damage caused by these stresses, is evidenced by the fact that 22% (1,184 of 5,384) of the upregulated DEGs identified in the present work were also upregulated after exposure to early drought stress in our previous work (Barratt et al., 2023b), and by the observations of key heat stress-responsive gene families, such as HSPs, acting to enhance tolerance to drought and salinity stresses, as well as heat, in other species (Gao et al., 2012; Li et al., 2016; Zhai et al., 2016; Guo et al., 2020; Jiang et al., 2020; Rahman et al., 2022; Do et al., 2023).

### *TaMAPKKK18-like* and *TaERD15-like* may coordinate a transcriptional shift away from growth and the response to abiotic stresses other than heat

Given that the downregulation of genes likely involved in responding to abiotic stresses other than heat seems to be a substantial constituent of the transcriptional heat stress response in wheat landraces, it was interesting that two of the hub genes identified in the co-expression network were connected to a large number of such downregulated DEGs in their respective modules, with one itself likely playing a role in the cold stress response and cold tolerance. The black module contained almost twice as many DEGs as expected (expected number = 216, observed number = 429, *p* = 1.82E-52) and was enriched in the “Response to abiotic stimulus” and “Response to water” GO terms (FDR-adjusted *p* = 0.004 and 0.019, respectively). *TraesCS1B02G384900*, *TaMAPKKK18-like*, was downregulated under heat stress (log2FC = -2.98, **Figure 3A**) and identified as a hub gene within the black module, being connected to 428 DEGs (100% of the remaining DEGs in the module). Meanwhile, the pink module was identified as particularly associated with the heat stress response as it was significantly enriched in DEGs (expected number = 197, observed number = 879, *p* = 0), whilst also being enriched in the GO terms “photosynthesis” and “cellular response to stimulus” (*p* = 1.5E-33 and 0.001, respectively). The hub gene in the module was *TraesCS3B02G409300*, *Triticum aestivum EARLY RESPONSIVE TO DEHYDRATION 15-like* (*TaERD15-like*). *TaERD15-like* was also found to be downregulated under heat stress (−2.52, **Figure 3B**), and was connected to 845 of the 879 remaining DEGs in the module (96%)—all of which were also downregulated.

*TaMAPKKK18-like*’s orthologue (identified *via* Ensembl Plants; Yates et al., 2022) in Arabidopsis, *AtMAPKKK18*, is a key part of ABA-mediated signal transduction, as it acts to phosphorylate proteins in an ABA-dependent manner (Matsuoka et al., 2015). This kinase activity can determine leaf senescence, growth, and stomatal dynamics, as overexpression of the gene led to smaller plants and increased leaf senescence of rosette leaves (Matsuoka et al., 2015), whereas knockout mutants showed more vigorous root growth, as well as increased stomatal aperture (Mitula et al., 2015)—suggesting a link with water use, and subsequently, the drought response. *TaERD15-like*’s orthologue in rice, *OsERD15*, is known to be both expressed more in cold-tolerant varieties and also to be induced during cold stress exposure (Sperotto et al., 2018; Rativa et al., 2020). Rice and wheat ERD15 proteins are relatively poorly characterised; however, in Arabidopsis, they are known to be integral players in the response to abiotic stress, mainly drought and cold, as they act as negative regulators of ABA signalling (Kariola et al., 2006; Aalto et al., 2012). In addition to a likely role responding to cold stress, which would be unrequired under high temperatures, a potential duty repressing ABA signalling may also explain the downregulation of the hub gene here, due to the key roles ABA plays during the heat stress response, including increasing antioxidant activity and sucrose metabolism, as well as upregulating the expression of HSPs and Hsfs (Li et al., 2020).

The likely involvement of both genes in ABA signal transduction, therefore, perhaps explains their identification as hub genes within their respective modules—as the expression of many genes in the wheat transcriptome will respond to this integral signal. *TaMAPKKK18-like*, however, may also be able to have far-reaching effects on gene expression thanks to connections to a suite of heat-responsive transcription factors and signalling proteins: 14% of the DEGs *TaMAPKKK18-like* was connected to in the module were transcription factors (from gene families such as MYB, WRKY, DREB, ERF, and Hsf), whereas *TaMAPKKK18-like* was also connected to 17 differentially expressed JAZ proteins—key repressors of JA-signalling and JA-induced gene expression (Santner and Estelle, 2007; Kazan and Manners, 2012; Wager and Browse, 2012; Sasaki-Sekimoto et al., 2014).

Within their respective modules, *TaMAPKKK18-like* and *TaERD15-like* were connected to a large number of DEGs which appear to be involved in responding to abiotic stresses other than heat. For instance, connected to *TaMAPKKK18-like* in the black module were as follows: homoeologues *TraesCS1A02G423800* (−2.25) and *TraesCS1B02G455900* (−2.51), *Triticum aestivum late embryogenesis abundant 14-A-like* genes, whose orthologue in Arabidopsis increased salt tolerance when overexpressed (Jia et al., 2014); *TraesCS6B02G268100* (−3.57) and its homoeologue *TraesCS6D02G238200* (−4.4), *Triticum aestivum AP2 domain CBF (CBFI)*, which are likely involved in the cold response (Medina et al., 1999); *TraesCS6D02G332500* (−2.58), *Triticum aestivum cold-shock CS120*, which is also likely involved in the response to cold stress thanks to shared sequence identity with regions of cold-response genes in Arabidopsis, such as *AtRAB18* (Lång and Palva, 1992; Lang et al., 1994; Mantyla et al., 1995; Puhakainen et al., 2004); and *TraesCS1D02G263200* (−1.87), *Triticum aestivum ERF019-like*, which encodes an ethylene-responsive transcription factor whose orthologue in Arabidopsis improves drought tolerance and water use, through reduced stomatal aperture and transpiration, when overexpressed (Scarpeci et al., 2017). Similarly, *TaERD15-like* was connected to *TraesCS4B02G332700* (−2.32), *TraesCS4B02G332800* (−2.68), *TraesCS4D02G329500* (−2.21), and *TraesCS5A02G503800* (−2.91)—copies of *Triticum aestivum ABA-inducible PHV A1-like*, also known as *HVA1* or *WCOR615*. The barley gene, *HVA1*, has been found to increase drought and salinity tolerance when overexpressed in rice and wheat (Xu et al., 1996; Sivamani et al., 2000; Rohila et al., 2002; Chandra Babu et al., 2004; Bahieldin et al., 2005; Chen et al., 2015). *TaERD15-like* was also connected to *TraesCS5A02G503900* (−1.58), *Triticum aestivum cold-responsive LEA/RAB-related COR (Wrab17.1)*, another COR protein which has been shown to respond to ABA and cold stress (Tsuda et al., 2000), and may play a role in the biotic stress response (Gaoshan et al., 2018).

Despite both appearing to be involved in determining the downregulation of superfluous drought- and cold-responsive genes under early heat stress, *TaMAPKKK18-like* and *TaERD15-like* may also play key roles in the regulation of other genes involved in separate processes. For instance, reduced expression of *TaMAPKKK18-like* under heat stress may also activate stress hormone signalling, thanks to the co-downregulation of several ABA and JA signalling repressors such as *TraesCS7A02G201200* (−5.43), *TaTIFY 11e-like*, encoding a likely repressor of jasmonate responses due to its membership in the JAZ family (Santner and Estelle, 2007; Kazan and Manners, 2012; Wager and Browse, 2012; Sasaki-Sekimoto et al., 2014); and *TraesCS3A02G347500* (−2.66), *Triticum aestivum WRKY24-like*, whose orthologue in rice is a negative regulator of GA and ABA signalling (Zhang et al., 2015). The downregulation of such signalling genes, therefore, may allow key stress hormones to accumulate in plant tissue under heat stress and act as part of the heat stress response (Li et al., 2020).

Similarly, *TaERD15-like* is itself a likely repressor of ABA signalling downregulated under early heat stress; however, we found that it was connected to a large number of genes which seemingly play roles in photosynthesis, largely as part of PSII—the most heat-labile part of the photosynthetic apparatus (Yamamoto, 2016; Wang et al., 2018; Hu et al., 2020). Nine of these downregulated genes, *TraesCS1A02G403300* (−2.79), *TraesCS1B02G432700* (−2.73), *TraesCS1D02G411300* (−2.66), *TraesCS2A02G204800* (−1.87), *TraesCS2B02G220100* (−1.53), *TraesCS5B02G463100* (−3.06), *TraesCS5D02G329200* (−2.61), *TraesCS5D02G464900* (−3.9), *TraesCS7D02G276300* (−2.53), encode Chlorophyll a-b binding proteins, which form antenna complexes in PSII and act to absorb sunlight, transferring excitation energy to PSII to power photosynthetic electron transport (Jansson, 1994; Jansson, 1999). Under intense heat stress, PSII light-harvesting complexes fall off of thylakoid membranes, subsequently reducing the efficiency of electron transfer, which results in reduced photosynthesis (Janka et al., 2013; Mathur et al., 2014; Hu et al., 2020). There were 16 other DEGs connected to the hub that were constituent parts of the heat-labile PSII reaction centre, all of which were also downregulated under heat stress. The downregulation of these genes, as well as the nine Chlorophyll a-b binding protein genes, suggests inactivation of PSII under heat stress. As well as being connected to genes which are part of PSII, *TaERD15-like* was also connected to *TraesCS4A02G177500* (−2.15), *Triticum aestivum ribulose bisphosphate carboxylase/oxygenase activase A, chloroplastic-like*—otherwise known as *TaRca2* (Caruana et al., 2022). The TaRca2 isoforms are the most heat-labile of the Rubisco activase proteins; meaning, during periods of heat stress, less functional protein is available to remodel the active site of Rubisco to release tightly bound inhibitors, leading to a reduced photosynthetic rate (Salvucci et al., 1985; Bhat et al., 2017; Degen et al., 2021).

The damaging effect of heat stress exposure on PSII activity is well known (Yamamoto, 2016; Wang et al., 2018; Hu et al., 2020); however, here we see evidence that *TaERD15-like* may be playing a central role in this inactivation, as it was connected to a large number of downregulated PSII genes. Downregulation of *TaERD15-like*, therefore, may be a preventative tactic taken by plants to limit the build-up of damaged photosynthesis proteins under heat stress—a wise tactic considering that this is often toxic to cells (McClellan et al., 2005; Gil et al., 2017).

### sHSP hub gene may promote the heat stress response *via* upregulation of thermoprotectants and stress-responsive transcription factors

The blue module was enriched in DEGs (expected number = 610, observed number = 966, *p* = 6.34E-52), but not in any GO terms related to the stress response. The most well-connected gene in this module, *TraesCS4D02G212300*, *Triticum aestivum 17.9 kDa class I heat shock protein-like* (LOC123097951), was also upregulated under heat stress (3.29, **Figure 3C**). The small HSP (sHSP) hub gene also shares remarkable sequence identity (97%) with *TaHSP26*; a sHSP located in the chloroplast, whose expression is induced by heat stress exposure (Chauhan et al., 2012; Khurana et al., 2013). *TaHSP26* has been known to be involved in thermotolerance for over two decades with Joshi et al. (1997) finding that the gene was expressed in thermotolerant recombinant inbred lines, but not susceptible ones. More recently, further evidence for the gene’s role in increasing thermotolerance has been provided, as when the gene was expressed in *Arabidopsis*, PSII activity, photosynthetic pigment production, biomass, and seed yield under heat stress were all higher than that of WT plants (Chauhan et al., 2012). The present work suggests that the similar gene, and fellow sHSP, *TraesCS4D02G212300* (herein referred to as the “sHSP hub gene”), may act as a key coordinator of the response to early heat stress.

The sHSP hub was connected to 952 of the 966 remaining DEGs in the module, 60 of which were annotated as HSPs. For example, *TraesCS1D02G284000* (2.99), *TaHSP70d*, is a known thermotolerance gene (Hu et al., 2018); *TraesCS2A02G033700* (2.44), *Triticum aestivum heat shock protein 90-1*, shows sequence identity (76%) to *AtHSP81.4*, whereas *TraesCS1A02G340100* (4.87), and homeologues *TraesCS3B02G308100* (5.82) and *TraesCS3D02G273600* (3.6), encode *Triticum aestivum* chaperone protein ClpB1-like and share sequence identity (71%, 72%, and 72%, respectively) with *AtHSP101* (*ClpB1*)—a gene whose expression is known to respond to heat stress, and whose protein aids protein refolding under high temperatures, and facilitates the deaggregation of toxic ubiquitylated protein aggregates *via* interaction with the proteasome (Queitsch et al., 2000; Hong and Vierling, 2001; Tonsor et al., 2008; McLoughlin et al., 2019). The sHSP hub gene was also connected to six genes: *TraesCS1B02G294300* (4.02), *TraesCS1A02G285000* (2.93), *TraesCS3B02G390700* (4.32), *TraesCS3D02G351900* (3.85), *TraesCS3D02G352400* (2.0), and *TraesCS4A02G098600* (3.58), which share 78% sequence identity with *AtHSC70-1*—a repressor of thermotolerance in Arabidopsis (Tiwari et al., 2020). The upregulation of these six genes, and our previous observation that increased expression of a wheat orthologue of *AtHSC70-1* can be used as a marker for increased thermotolerance (Barratt et al., 2023a), suggests these genes may play positive roles in both thermotolerance and the heat stress response in *T. aestivum*. The majority of the 60 HSPs connected to the hub were fellow sHSPs, a group of proteins known to delay formation of harmful protein aggregates under heat stress and enhance thermotolerance in a wide variety of plant species, such as rice, maize, and poplar (Murakami et al., 2004; Kim et al., 2012; Sun et al., 2012; Zhou et al., 2012; Chen et al., 2014; Merino et al., 2014; Tian et al., 2021). The upregulation of these genes under heat stress in wheat corroborates these observations and suggests that the hub gene may act as a regulator of these crucial protective genes.

The sHSP hub was also connected to a large group of transcription factors known to play key roles in abiotic stress responses in wheat and other species. Expression of homoeologues *TraesCS7A02G270100* (2.05) and *TraesCS7B02G168300* (2.08), *TaHsfB2-3* and *TaHsfB2-4*, respectively, respond to heat stress treatment (Duan et al., 2019), and these genes belong to a family of transcription factors which act to determine the expression of many other stress-responsive gene family members as part of abiotic stress responses, particularly HSPs (Guo et al., 2016). *TraesCS3A02G281900* (1.62), *Triticum aestivum probable WRKY transcription factor 65*, shows some sequence identity (74%) to a small region of its Arabidopsis orthologue, *AtWRKY65*—a gene known to increase thermotolerance and repress thermomorphogenesis when acting alongside its homologues (Qin et al., 2022). However, *TraesCS2D02G414300* (2.72), *Triticum aestivum ethylene-responsive transcription factor ERF105-like*, may act as part of other stress responses. The gene’s orthologue in Arabidopsis is involved in promoting freezing tolerance and cold acclimation (Bolt et al., 2017), suggesting an action as part of the generalised stress response in wheat, as opposed to a tailored response to cold stress. Similarly, the hub is connected to other genes which have previously been described as playing roles in response to stresses other than heat, but their upregulation, and connection to the hub gene, in the present work suggests they may act as part of the general stress response: *TraesCS4B02G176700* (2.03), *TaWRKY19*, has been shown to regulate abiotic stress tolerance when overexpressed in Arabidopsis—leading to increased salt, drought, and freezing tolerance, likely *via* the upregulation of stress-responsive genes such as *DREB2A*, *RD29A*, *RD29B*, and *Cor6.6* (Niu et al., 2012), whereas *Aegilops tauschii subsp. strangulata ethylene-responsive transcription factor 1-like* (*TraesCS4B02G200200*; 1.8) has been shown to prevent disease progression and regulate the expression of genes involved in the biotic stress response (Lorenzo et al., 2003). Likewise, *TraesCS5D02G148800* (4.58), *TaNAC29*, increases salt and drought tolerance when overexpressed in Arabidopsis (Huang et al., 2015), whereas overexpression of the Arabidopsis namesake of *TraesCS5A02G510100* (2.86) *Triticum aestivum zinc finger protein CONSTANS-LIKE 4-like*, *AtCOL4*, led to reduced ABA sensitivity and increased salinity tolerance (Min et al., 2015). The upregulation of such genes in the present work suggests a shared role as part of the general stress response, unlike the downregulated drought-, salinity-, and cold-responsive genes connected to *TaMAPKKK18-like* and *TaERD15-like* which likely act as part of the tailored response to these stresses. The hub’s connection to this suite of upregulated transcription factors, as well as its connection to 60 fellow upregulated HSPs and sHSPs, suggests a new function for the poorly characterised sHSP hub gene as a potential activator of the heat stress response.

Although the role of sHSPs, and TaHSP26 in particular, in acquired thermotolerance and the response to heat stress is widely accepted to be the prevention of protein misfolding and aggregation of heat labile proteins, work by Guihur et al. (2020) suggests that this group of proteins may also act to regulate the activity of signalling proteins, which, in turn, improves thermotolerance by impeding processes such as cell death (Guihur et al., 2020). It may be possible, therefore, that the sHSP hub gene identified in the present work regulates the heat-responsive expression of the genes it is connected to in the co-expression network *via* effects on the activity of these signalling proteins; however, further work is required to determine the exact mechanism by which the sHSP hub gene may indeed regulate the expression of these genes in response to heat stress.

In the present work, we have demonstrated that early heat stress exposure causes large shifts in the wheat transcriptome, with almost 8,000 response DEGs being identified, whereas the likely functionalities of genes being upregulated and downregulated suggests a shift away from growth and development, to stress response and damage mitigation. We also observed the widespread downregulation of genes potentially involved in responding to other abiotic stresses, likely due to the fact that the cellular conditions these genes respond to are not present under heat stress. We then paired these data with the co-expression network to identify heat-associated modules, within which were several promising candidates which may act as regulators of the transcriptional and physiological early heat stress response. Downregulation of two of the most promising candidates under early heat stress (*TaMAPKKK18-like* and *TaERD15-like*) may act to downregulate the expression of these superfluous stress-responsive genes, whereas a sHSP hub gene may activate the expression of HSPs and fellow sHSPs, as well as transcription factors known to play key roles in various abiotic stress responses, including the response to heat. This work, therefore, represents a vital step toward the creation of more thermotolerant wheat varieties, and provides key new insights into the transcriptional response of wheat to early heat stress, as well as candidate genes which may regulate this response.

## Data availability statement

The datasets presented in this study can be found in online repositories. The names of the repository/repositories and accession number(s) can be found below: NCBI gene expression omnibus (GEO) under accession number GSE232367.

## Author contributions

LB and AH conceived and planned the project. LB performed plant growth experiments and RNA extraction. SFO performed transcriptome data mapping and QPCR. LB conducted transcriptomic analyses. LB and SFO wrote the manuscript, and all authors reviewed and edited it. All authors contributed to the article and approved the submitted version.

## Supplementary material

Supplementary Figure 1 Gene Ontology enrichment analysis of DEGs in graphical format: **(A)** Up-regulated genes – Biological Process; **(B)** Up-regulated genes – Cellular Component; **(C)** Up-regulated genes – Molecular Function; **(D)** Down-regulated genes – Biological Process; **(E)** Down-regulated genes – Cellular Component; **(F)** Down-regulated genes – Molecular Function.
