# Supplementary figures and images for: Identification of candidate regulators of the response to early heat stress in climate-adapted wheat landraces via transcriptomic and co-expression network analyses
Source: Front Plant Sci. 2024 Jan 3;14:1252885. doi: 10.3389/fpls.2023.1252885 (PMC10791870; doi:10.3389/fpls.2023.1252885)

[illegible]

b

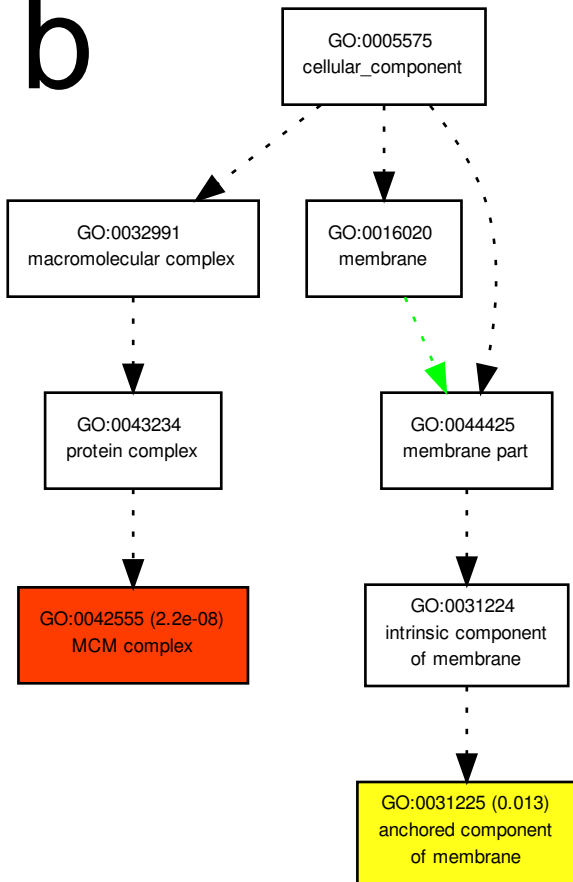

C

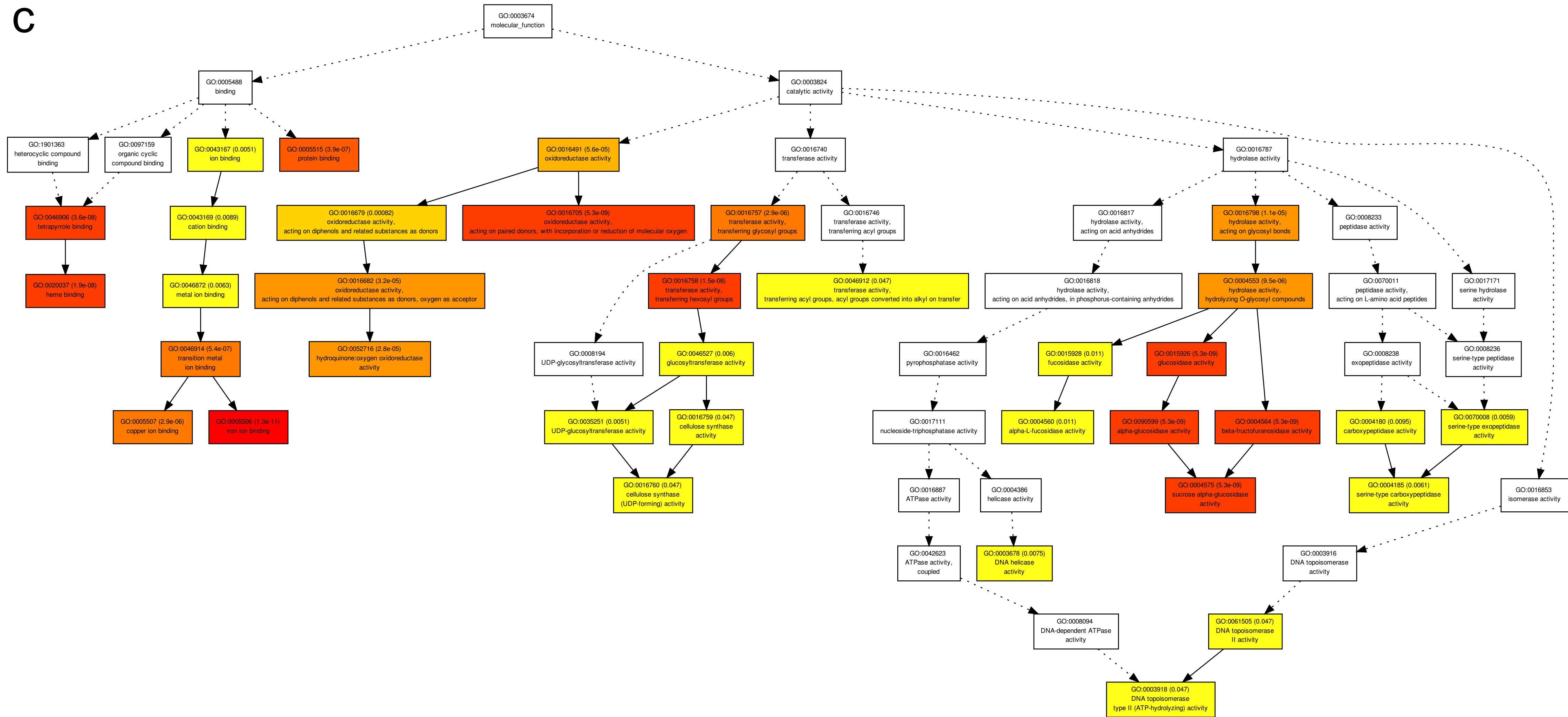

d

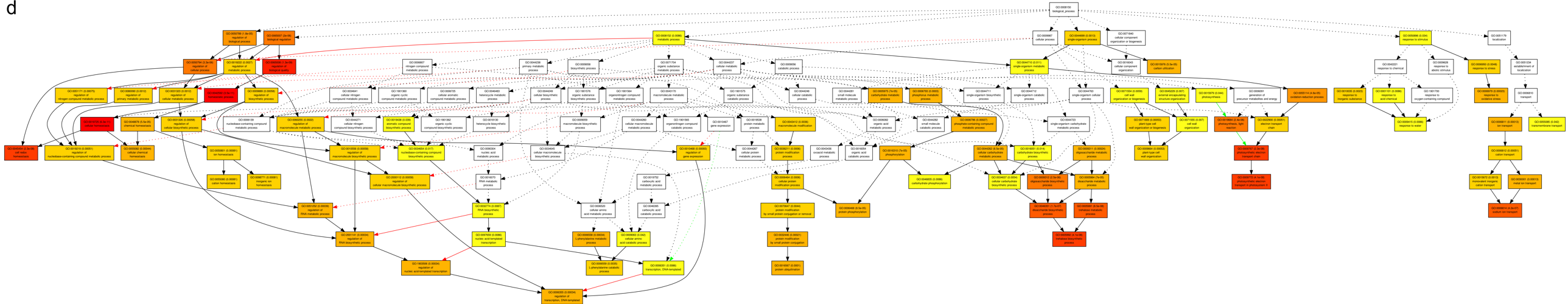

e

GO:0005575  
cellular\_component

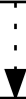

GO:0005576 (5.2e-08)  
extracellular region

f

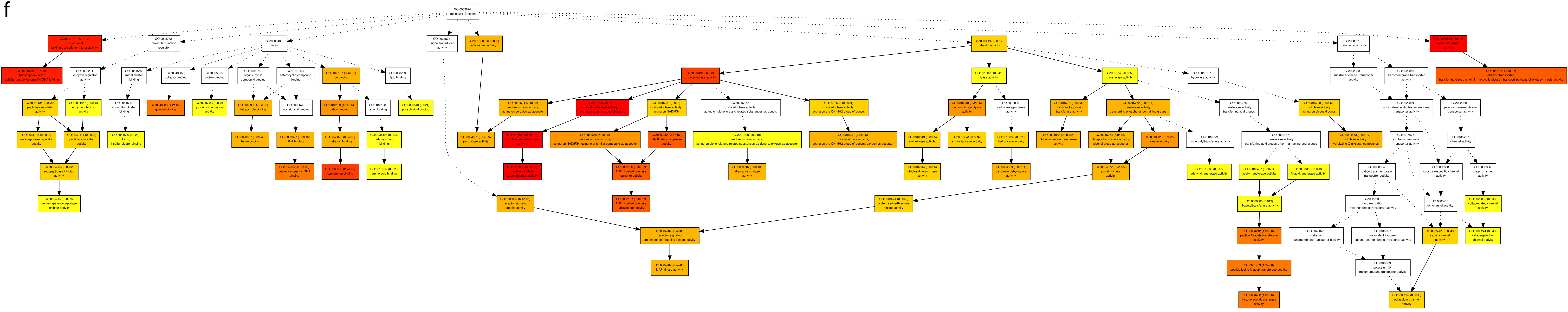

Supplement: Supplementary Figure 1 — Gene Ontology enrichment analysis of DEGs in graphical format: (A) Up-regulated genes – Biological Process; (B) Up-regulated genes – Cellular Component; (C) Up-regulated genes – Molecular Function; (D) Down-regulated genes – Biological Process; (E) Down-regulated genes – Cellular Component; (F) Down-regulated genes – Molecular Function. [file DataSheet_1.pdf]
